# Patient-specific optimisation of administered activity and acquisition times for ^18^F-FDG PET imaging

**DOI:** 10.1186/s13550-016-0250-3

**Published:** 2017-01-13

**Authors:** Fred Wickham, Helena McMeekin, Maria Burniston, Daniel McCool, Deborah Pencharz, Annah Skillen, Thomas Wagner

**Affiliations:** 1 Nuclear Medicine Department, Royal Free London NHS Foundation Trust, Pond Street, London, NW3 2QG UK; 2 Hermes Medical Solutions, 22 Long Acre, London, WC2E 9LY UK

**Keywords:** Positron emission tomography, Administered activity, Acquisition time, Optimisation, Image quality, Noise equivalent counts

## Abstract

**Background:**

The purpose of this study is to identify a method for optimising the administered activity and acquisition time for ^18^F-FDG PET imaging, yielding images of consistent quality for patients with varying body sizes and compositions, while limiting radiation doses to patients and staff. Patients referred for FDG scans had bioimpedance measurements. They were injected with 3 MBq/kg of ^18^F up to 370 MBq and scanned on a Siemens Biograph mCT at 3 or 4 min per bed position. Data were rebinned to simulate 2- and 1-min acquisitions. Subjective assessments of image quality made by an experienced physician were compared with objective measurements based on signal-to-noise ratio and noise equivalent counts (NEC). A target objective measure of image quality was identified. The activity and acquisition time required to achieve this were calculated for each subject. Multiple regression analysis was used to identify expressions for the activity and acquisition time required in terms of easily measurable patient characteristics.

**Results:**

One hundred and eleven patients were recruited, and subjective and objective assessments of image quality were compared for 321 full and reduced time scans. NEC-per-metre was identified as the objective measure which best correlated with the subjective assessment (Spearman rank correlation coefficient 0.77) and the best discriminator for images with a subjective assessment of “definitely adequate” (area under the ROC curve 0.94). A target of 37 Mcount/m was identified. Expressions were identified in terms of patient sex, height and weight for the activity and acquisition time required to achieve this target. Including measurements of body composition in these expressions was not useful. Using these expressions would reduce the mean activity administered to this patient group by 66 MBq compared to the current protocol.

**Conclusions:**

Expressions have been identified for the activity and acquisition times required to achieve consistent image quality in FDG imaging with reduced patient and staff doses. These expressions might need to be adapted for other systems and reconstruction protocols.

**Electronic supplementary material:**

The online version of this article (doi:10.1186/s13550-016-0250-3) contains supplementary material, which is available to authorized users.

## Background

The aim of this study was to identify a method for optimising the administered activity and acquisition time for fluorine-18 fluorodeoxyglucose (^18^F-FDG) positron emission tomography (PET) imaging for individual patients to ensure that images of consistent quality are produced for patients with varying body sizes and compositions, while limiting radiation doses to patients and staff. National and international limits and guidelines should be taken into consideration, and acquisition times should be practical.

The detectability of low-contrast features in PET scans depends on count statistics, which in turn depend on various factors including the efficiency of the scanner, administered activity, uptake time, acquisition time and the size of the patient. Advances in PET hardware and software in the last two decades have led to significant increases in the sensitivity of PET scanner systems [1]. In PET imaging, the relationship between the administered activity and the count statistics can be characterised by a plot of noise equivalent counts (NEC) against activity [2]. Increasing the activity up to the peak in the noise equivalent count curve will improve the count statistics and the detectability of low-contrast features but will also increase the radiation dose to the patient and to the staff involved in administering the activity and scanning the patient. The NEC can also be improved by increasing the acquisition time, but this will be limited by the amount of time for which the patient can be expected to remain still on the scanner couch and also by workload considerations on the scanner. Noise equivalent count rates tend to be lower for heavier patients [3], and it has been demonstrated that even if activity is administered in proportion to the patient’s weight, image quality is lower in heavier patients [4, 5]. This effect may be mitigated by further increasing the administered activity or by increasing the acquisition time for heavier patients. However, increasing the administered activity has been shown to be less effective at improving image quality than the same proportional increase in acquisition time [5].

In the UK, the Administration of Radioactive Substances Advisory Committee (ARSAC) specifies a diagnostic reference level (DRL) of 400 MBq for ^18^F-FDG whole-body tumour imaging [6] but does not give any guidance on the modification of administered activity according to scanner type or patient size. The Society of Nuclear Medicine and Molecular Imaging (SNMMI) Guidelines on Tumor Imaging with ^18^F-FDG PET/CT [7] suggests that adults are administered between 370 and 740 MBq. Other guidelines have moved to modifying administered activities and acquisition times according to the size of the patient [8, 9].

At this centre, patients are administered with 3 MBq per kg of body weight up to a maximum of 370 MBq. Most patients are scanned for 3 min per bed position. This is increased to 4 min for patients with a body mass index (BMI) greater than 30.

An approach to optimising the administered activity advocated by Watson et al. [3], Lartizien et al. [10] and Inoue et al. [11] is to identify the activity required to achieve a noise equivalent count rate equal to or close to the peak value. Using this activity, the required image quality may be achieved in the lowest acquisition time. However, this approach is not consistent with the requirement that radiation doses are kept as low as reasonably practicable (ALARP); it may be possible to achieve the same image quality with lower radiation doses to the patient and staff by using a lower activity and increasing the scan duration, particularly for smaller patients [12].

The impact of a reduction of acquisition time on image quality or lesion detectability can be investigated by acquiring data in list mode, so that the raw data can be rebinned to simulate scans acquired with reduced acquisition times [13–15].

The approach to optimisation adopted by de Groot et al. [4], by Accorsi et al. [12] and in this study is to identify a target value for an objective measure of image quality and then to identify the activity required to achieve that target in a practical scan duration for patients of different sizes.

Signal-to-noise ratio (SNR) in regions where uptake might be expected to be uniform such as the liver and aortic arch is a commonly used measure of image quality in studies investigating the relationship between image quality and administered activity [4]. Other studies have suggested that measures based on NEC may coincide better with physicians’ subjective assessment [9, 16, 17].

The study was split into three phases:

1. Identifying an objective measure of image quality which gives good agreement with a physician’s subjective assessment and a target value for this measure
2. Identifying a combination of administered activity and acquisition time required to achieve this target for each subject
3. Identifying expressions in terms of measurable patient characteristics which could predict the administered activity and acquisition time required

Previous studies have included one or two of these phases, but to our knowledge, there are no studies which have included all three.

## Phase 1: identifying a target objective measure of image quality

### Method

#### Subjects

The study was approved by the National Research Ethics Service Committee London – Hampstead (REC reference: 14/LO/1027). The subjects were patients referred to the centre for a whole-body ^18^F-FDG PET scan for a range of indications between September 2014 and June 2015. All subjects gave informed written consent.

#### Measurements

The subjects were scanned on a Siemens Biograph mCT scanner (Knoxville, TN, USA) in list mode using the centre’s current protocol (see Table 1). Bioimpedance measurements of each subject’s body composition were also made using an InBody S20 Body Composition Analyser (Seoul, Korea) for use in phase 3 of the study.

**Table 1:** Current scanning and reconstruction protocol

Scanner	Siemens Biograph mCT with an extended axial field of view
Activity administered	3 MBq per kg of body weight up to a maximum of 370 MBq
Uptake time	60 min
Time per bed position	3 min increased to 4 min for BMI greater than 30
Bed overlap	42%
Attenuation correction	CT
Reconstruction method	TrueX + TOF (UltraHD-PET)
Iterations/subsets	2/21
Filter	Gaussian 2 mm FWHM
Matrix	200 × 200 × 75
Voxel dimensions (mm)	4.1 × 4.1 × 3.0

Scan data were rebinned to simulate 2 and 1 min per bed position acquisitions. All data were reconstructed using the centre’s current protocol (see Table 1).

#### Objective measures of image quality

For each reconstruction, the following objective measures of image quality were calculated using methods described by Fukukita et al. [9]:

SNR was measured in the liver (SNR_liver_) and aortic arch (SNR_AA_) using Hermes HybridViewer software (Hermes Medical Solutions, Stockholm, Sweden). For SNR_liver_, a spherical volume of interest (VOI) with a diameter of 50 mm was positioned in the centre of the right lobe of the liver, avoiding the major blood vessels and any lesions. For SNR_AA_, a VOI with a diameter of 15 mm was positioned in the centre of the aortic arch. The SNR in each VOI was calculated as the ratio of the mean to the standard deviation of the voxel value.

NEC measurements were made using data from bed positions covering the subject’s torso from above the top of the bladder to below the shoulders. Bed positions including the bladder were excluded as true coincidences from uptake in the bladder would increase the NEC but are unlikely to make a useful contribution to the image. NEC was calculated according to Eq. 1:

1 $$ \mathrm{N}\mathrm{E}\mathrm{C}={\left(1-SF\right)}^2\frac{{\left(P-D\right)}^2}{P-D+\left(1+k\right)\times D\times \raisebox{1ex}{$a$}\!\left/ \!\raisebox{-1ex}{$A$}\right.} $$

*SF* is the scatter fraction, *P* is the prompt counts, *D* is the delayed counts, *k* is the random scaling factor, *a* is the axial cross-sectional area of the subject’s torso and *A* is the axial cross-sectional area of the PET field of view.

*SF*, *P* and *D* were extracted from the DICOM header files for the reconstructed and raw PET data. *k* was set to 1, as randoms were estimated from the delayed window. *a* was measured at the height of the top of the liver on CT images; *a* was the area of the region of interest corresponding to the patient cross section drawn using the thresholding tool in Hermes HybridViewer.

It should be noted that Eq. 1 differs from the expression used by Fukukita et al. [9] for calculating NEC in patient scans. In [9], the expression used for calculating NEC in phantom acquisitions includes the phantom cross-sectional area term, $$ \raisebox{1ex}{$a$}\!\left/ \!\raisebox{-1ex}{$A$}\right. $$, in the denominator but the expression for calculating NEC in patient acquisitions does not. In this study, this term has been included in the expression used for calculating NEC in patients so that the NEC calculated reflects the noise equivalent counts attributable to the patient rather than the entire field of view.

Noise equivalent counts per unit length (NEC_patient_) was calculated by dividing NEC by the total axial length of the scan used to calculate NEC.

Noise equivalent count density (NEC_densit*y*_) was calculated by dividing NEC_patient_ by *a*.

#### Subjective assessment of image quality

All the reconstructed data were anonymized and presented to an experienced nuclear medicine physician in random order. Subjective assessments of image quality were made using a five-point scale: 1, seriously inadequate; 2, inadequate; 3, marginally adequate; 4, definitely adequate; and 5, more than adequate.

#### Statistical analysis

Subjective assessments of image quality were compared with the objective measurements using Stata 11.1 Statistics/Data Analysis software (StataCorp LP. College Station, TX). Correlation coefficients for Spearman rank correlation of subjective against objective measures of image quality and the areas under receiver operating characteristic (ROC) curves describing the ability of objective measures to identify images with a subjective assessment of definitely adequate or better were calculated.

### Results

Measurements were made for 111 subjects. Subject characteristics are summarised in Table 2. Thirty-two (29%) subjects had a BMI greater than 30 and were scanned for 4 min per bed position. The mean administered activity was 231 MBq. For six subjects, PET data were not acquired in list mode and could not be rebinned to simulate reduced time acquisitions. For two subjects, raw PET data were not saved correctly and NEC values could not be calculated. In total, 321 full count and reduced count scans were reconstructed. SNR_liver_, SNR_AA_ and subjective image quality were assessed in all of these. NEC calculations were possible for 317 scans.

**Table 2:** Characteristics of subjects

Variable	Number (percent)/range (median)
Number of subjects	111
Number of females	52 (47)
Age (years)	23–89 (66)
Weight (kg)	35–139 (76)
Height (m)	1.45–1.97 (1.67)
Reason for referral:	
Oncology	70 (63)
Haematology	26 (23)
Inflammation/infection	15 (14)

No scans were identified as having image quality more than adequate.

Correlation coefficients for Spearman rank correlation of subjective against objective measures of image quality and the areas under ROC curves describing the ability of objective measures to identify images with a subjective assessment of definitely adequate or better are given in Table 3. NEC_patient_ is the objective measure of image quality which gives the best agreement with the subjective assessment according to both statistics. The difference between the Spearman rank correlation coefficient for NEC_patient_ and for SNR_AA_ is statistically significant at the 95% confidence level, but for SNR_liver_ and NEC_density_, the correlation coefficients are not significantly different from the value for NEC_patient_. The area under the ROC curve for NEC_patient_ is statistically significantly higher than the area under the ROC curves for the other measures.

**Table 3:** Spearman rank correlation coefficients and areas under ROC curves showing agreement between objective and subjective assessments of image quality

Objective measure	Spearman rank correlation coefficient			Area under the ROC curve for identifying images which are “definitely adequate” or better		
	Value	95% confidence interval	*p* value for difference from NEC_patient_	Value	95% confidence interval	*p* value for difference from NEC_patient_
SNR_liver_	0.74	0.68–0.78	0.43	0.90	0.86–0.93	0.03
SNR_AA_	0.60	0.52–0.66	<0.01	0.82	0.77–0.86	<0.01
NEC_patient_	0.77	0.72–0.81		0.94	0.91–0.96	
NEC_density_	0.73	0.68–0.78	0.35	0.91	0.88–0.94	0.01

**Fig. 1:**
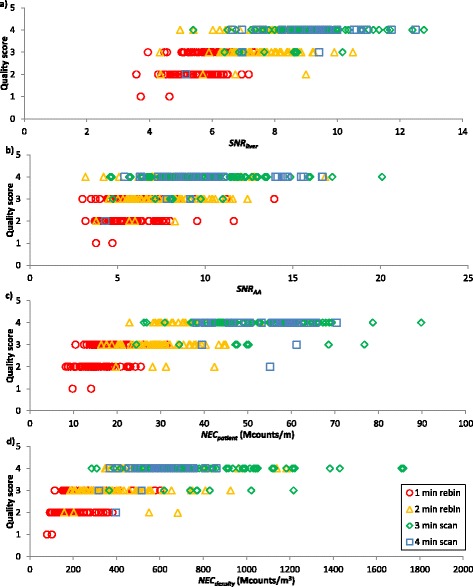
Subjective quality score plotted against **a** SNR_liver_, **b** SNR_AA_, **c** NEC_patient_, **d** NEC_density_

**Fig. 2:**
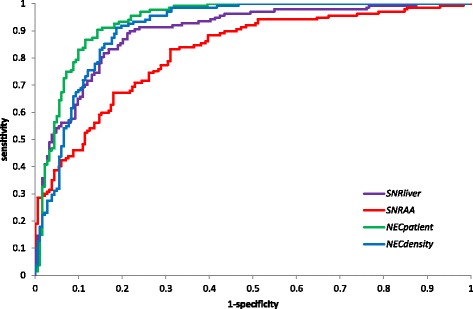
ROC curves for identifying images which are “definitely adequate” or better for each of the objective measures

Figure 1 shows the subjective assessment score plotted against SNR_liver_, SNR_AA_, NEC_patient_ and NEC_density_. Figure 2 shows ROC curves for each of the objective measures. Subjective and objective measures of image quality for each subject are available in Additional file 1.

Based on these results, a target value for NEC_patient_ of 37 Mcounts/m was identified. Ninety percent of scans with a subjective image quality assessment of marginally adequate or worse have values for NEC_patient_ of less than 37 Mcounts/m. Eighty-one percent of the scans identified as definitely adequate or better have values for NEC_patient_ of more than 37 Mcounts/m. Eighty-six percent of scans with NEC_patient_ of more than 37 Mcounts/m are definitely adequate or better.

## Phase 2: identifying the administered activity and acquisition time required to achieve the target

### Method

For each subject, a method described by Watson et al. [3] was used to derive an expression for noise equivalent count rate (NECR) against the administered activity.

The National Electrical Manufacturers Association test for the measurement of scatter fraction, count losses, and randoms was carried out by repeated scanning of a phantom filled with ^18^F as described in NEMA NU 2-2012 Performance Measurements of Positron Emission Tomographs [18]. For each scan of the phantom, activity in the phantom was calculated and singles rate, prompt rate and delayed count rate were extracted from the DICOM header files. These were used to express activity, prompt rate and delayed count rate as object-independent functions of the singles rate in the form of degree 6 polynomials.

For each subject scan, the object-independent functions were scaled according to the values for the activity, prompt rate and delayed count rate recorded at the singles rate of the subject scan. The scaled polynomials were combined with measured values for the scatter fraction and subject cross-sectional area using Eq. 1 to generate an expression for noise equivalent count rate against the administered activity for each subject.

These expressions were used to calculate the injected activity required to achieve the target NEC_patient_ at 3 and 4 min per bed position.

Full details of the method used to derive expressions for NECR against the administered activity are given by Watson et al. [3]. A useful explanation of how these can be used to identify suitable administered activities and acquisition times is given by Accorsi et al. [12].

### Results

For two subjects, the raw PET data were not saved correctly, so NECR could not be calculated. Plots of NECR against the administered activity were obtained for 109 subjects.

**Fig. 3:**
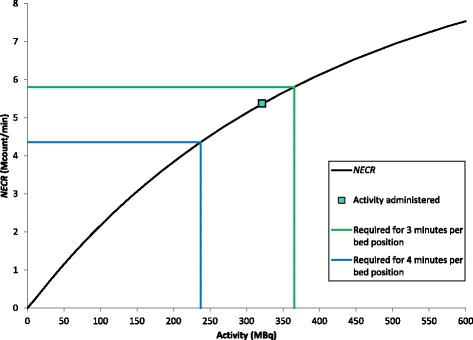
An example of a plot of NECR against activity for a subject, showing the activity administered and the NECR and activity required to achieve the target value for NEC_patient_ at 3 and 4 min per bed position

Figure 3 shows an example of a plot of NECR against an activity for a subject, with the activity administered and the activity required to achieve the target value at 3 and 4 min per bed position shown. The subject was administered 321 MBq and scanned for 4 min per bed position. The NECR was 5.37 Mcounts/min. The length scanned by the bed positions included was 0.47 m, so the NEC_patient_ was 45.7 Mcount/m (5.37 × 4/0.47). To achieve the target of 37 Mcount/m in 4 min requires an NECR of 4.35 Mcount/min (37 × 0.47/4) which corresponds to an administration of 237 MBq. To achieve the target in 3 min requires an NECR of 5.80 Mcount/min (37 × 0.47/3) which corresponds to an administration of 365 MBq.

## Phase 3: predicting the required administered activity and acquisition time in terms of measurable patient characteristics

Multiple regression analysis was used to identify expressions that could be used to determine whether patients should be scanned at 3 or 4 min per bed position and the activity required to achieve the target NEC_patient_ in terms of measurable characteristics for each patient.

### Method

The characteristics investigated were sex, age, height, weight and the measurements of body composition generated by the InBody S20 Body Composition Analyser. These included intracellular water, extracellular water, total body water, protein, soft lean mass, fat free mass, body fat mass, skeletal muscle mass, percent body fat, water of trunk, edema score, visceral fat area, obesity degree, body cell mass, bone mineral contents, basal metabolic rate and fat mass of trunk. Characteristics for each subject are available in Additional file 1.

Expressions for activity were sought in the form of Eq. 2. This allows for comparison with expressions included in the EANM Guideline [8].

2 $$ A=B\times C\times {D}^d\times {E}^e\times {F}^f\dots . $$

*A* is the activity required, *B* is a constant, *C* is a term whose value depends on the sex of the patient, *D*, *E*, *F*, etc. are continuous measures of other characteristics, such as age and weight, and *d*, *e*, *f*, etc. are powers to which those continuous measures are raised.

This was achieved by log-log regression, with the multiple regression tool of Stata 11.1 used to perform multiple linear regression of the logarithmic terms.

The correlation between height and weight meant that they could not both be investigated as individual terms in the same analysis. Instead, expressions were sought in terms of height, BMI and other variables. Once obtained, these were rearranged to give expressions in terms of height, weight and other variables. Similarly body composition measurements such as total fat mass or skeletal muscle mass were normalised by dividing by weight in order to reduce their correlation with height.

Goodness of fit was assessed using the adjusted *R* ^2^ value.

#### Outliers

Outliers were excluded before carrying out the analysis. A subject was excluded from the analysis if either:

1. The subjective assessment of image quality was definitely adequate, but the activity required to achieve the target NEC_patient_ in the time for which the patient was scanned was more than 60 MBq more than the activity actually administered. Four subjects were excluded on this basis.
2. The subjective assessment of image quality was marginally adequate or worse, but the activity required to achieve the target *NEC*_*patient*_ in the time for which the patient was scanned was more than 60 MBq less than the activity actually administered. Five subjects were excluded on this basis.

#### Procedure

Expressions for the activity required to achieve the target NEC_patient_ in 3 and 4 min per bed position were sought using all the subjects who were not excluded. Initially, the expressions were sought in terms of combinations of sex, age, height and BMI. The best combination of these was then combined with each of the body composition measures in turn to determine which of these was useful in improving the fit.

### Results

Expressions for the administered activity required to achieve the target activity were derived in terms of height, weight and sex. Inclusion of age or normalised measures of body composition in the analysis did not improve the correlation between the predicted and calculated values.

The expressions derived for administered activity and acquisition time required to achieve the target NEC_patient_ were as follows:

Patients for whom

3 $$ 0.176\times \left(1.13\ \mathrm{if}\ \mathrm{female}\right)\times \mathrm{height}{\left(\mathrm{m}\right)}^{-0.93}\times \mathrm{weight}{\left(\mathrm{kg}\right)}^{1.69}<250 $$

…administer

4 $$ 0.176\times \left(1.13\ \mathrm{if}\ \mathrm{female}\right)\times \mathrm{height}{\left(\mathrm{m}\right)}^{-0.93}\times \mathrm{weight}{\left(\mathrm{kg}\right)}^{1.69}\ \mathrm{MBq} $$

…and scan for 3 min per bed position.

Patients for whom

5 $$ 0.176\times \left(1.13\ \mathrm{if}\ \mathrm{female}\right)\times \mathrm{height}{\left(\mathrm{m}\right)}^{-0.93}\times \mathrm{weight}{\left(\mathrm{kg}\right)}^{1.69}>250 $$

…administer

6 $$ 0.219\times \left(1.12\ \mathrm{if}\ \mathrm{female}\right)\times \mathrm{height}{\left(\mathrm{m}\right)}^{-0.77}\times \mathrm{weight}{\left(\mathrm{kg}\right)}^{1.54}\ \mathrm{MBq} $$

…and scan for 4 min per bed position.

Expression (4) gives the activity required to achieve the target NEC_patient_ with an acquisition time of 3 min per bed position. If this is less than 250 MBq, then the patient is administered with this activity and scanned for 3 min per bed position. If expression (4) gives a value of more than 250 MBq, then the acquisition time is increased to 4 min per bed position and a reduced activity administered is calculated using expression (6).

The threshold of 250 MBq was chosen because it satisfies the requirement of being well below the diagnostic reference level of 400 MBq specified by ARSAC but is also practically achievable as it resulted in 10% of this patient group requiring scan times of 4 min per bed position. This is less than the 27% of these patients scanned for 4 min per bed position using the current procedure.

**Fig. 4:**
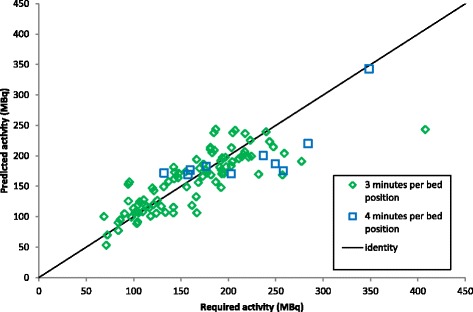
Predicted activities calculated using expressions (3) to (6) plotted against the activities required to achieve the target NEC_patient_ calculated in phase 2

Figure 4 shows the predicted activity to be administered to each subject according to expressions (3) to (6) plotted against the activity required to achieve the target NEC_patient_ calculated in phase 2

The mean activity which would have been administered to all subjects using this regime is 164 MBq. The mean activity actually administered was 230 MBq.

## Discussion

Noise equivalent counts per length of scan, NEC_patient_, has been identified as the objective measure of image quality which best coincides with the clinician’s assessment. This is consistent with the findings of McDermott et al. [16]. Mizuta et al. [17] concluded that noise equivalent count density NEC_density_ was a better measure, but this was based on 15 scans and it is not clear whether the difference was significant.

**Fig. 5:**
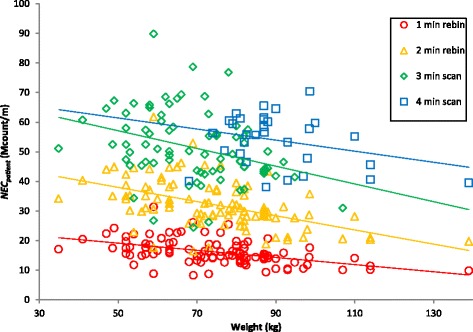
NEC_patient_ plotted against subject weight

Figure 5 shows a plot of NEC_patient_ against the subject weight. This demonstrates that even when the activity administered is scaled in proportion to the subject’s weight, as it was in this study, imaging quality tends to fall as weight increases.

Inspection of Fig. 1c might lead to the conclusion that the current protocol at this centre is reasonably successful. Using the protocol, 100 of the 111 full time scans (90%) were assessed as being definitely adequate. Reducing the acquisition time to 2 min per bed position reduces the proportion of definitely adequate scans to 37 out of 105 (35%), which suggests that the patients are not being scanned for much longer or administered much more activity than is needed to achieve adequate image quality.

An alternative explanation of these results is that the clinician tended to assess images of the quality that he was used to viewing as adequate and images with fewer counts than he was used to as less than adequate. This might also explain why no scans were identified as have image quality more than adequate. It might be useful to check that the clinician’s assessment of image quality coincided with the lesion detectability.

A target value for NEC_patient_ of 37 Mcounts/m was identified in this study. Omitting the patient cross-sectional area term from the NEC calculation would have reduced the calculated NEC by an average of 45%, so that the target of NEC_patient_ of 37 Mcounts/m is equivalent to 20 Mcounts/m if the patient cross-sectional area term is not included. This compares with the recommended targets of 13 Mcounts/m by Fukukita et al. [9], 14 Mcounts/m by Mizuta et al. [17] (after inclusion of an estimate of the scatter fraction) and 23 Mcounts/m by McDermott et al. [16]. It is unclear how Fukukita et al. identified their target value, as most of the scans included in their study which were assessed as “scarcely sufficient quality” or worse appear to have values for NEC_patient_ higher than this target. The Mizuta et al. target is based on only 15 scans. McDermott et al. justify their target value in terms of sensitivity and specificity, and we have taken a similar approach. However, it is important to recognise the limitations of using such terms in this context. Sensitivity and specificity are commonly used in clinical trials where they relate to representative samples taken from finite populations of subjects. In this case, the number of possible acquisitions and reduced acquisition time simulations which could have been performed is infinite. The apparent specificity achieved using the target value could have been improved by including simulations of acquisition times of much less than 1 min, a large proportion of which would have had poor image quality and a value of NEC_patient_ lower than the target. Similarly, the apparent sensitivity would have improved had the subjects been administered activities closer to those required to achieve the peak noise equivalent count rate and scanned for longer.

The discrepancy between the target values for NEC_patient_ identified by McDermott et al. and in this study may reflect the different scales used for the subjective image quality scoring, differences in expectations between the clinicians assessing the images, or the fact that the images were acquired on different scanners and reconstructed using different protocols.

As Accorsi et al. [12] have observed, NEC_density_ and NEC_patient_ are strictly measures of data quality, not image quality; they depend on the number of events recorded and the relative importance of random and scatter events, but not on how the data are reconstructed or processed. So, if the same raw data were reconstructed using a different reconstruction method, the resulting images might be of better or worse quality, but they would have the same NEC_patient_. This means that the target identified here for NEC_patient_ only applies to the reconstruction protocol used in this study. For example, in this study, data were reconstructed using time-of-flight (TOF). If TOF was not available, then a higher target NEC_patient_ might be required to ensure definitely adequate image quality. Conversely, the high-resolution reconstruction protocol use in this study includes point spread function modelling and relatively little smoothing. Use of a protocol which generated smoother images might require a lower target NEC_patient_ to achieve suitable noise characteristics.

Measures based on SNR, on the other hand, are direct measures of image quality. However, similar limitations apply. SNR calculated as the ratio of the mean to the standard deviation of the voxel value in a volume of interest can be improved by smoothing the data or by rebinning the data into larger voxels, but this will reduce the spatial resolution and will not necessarily improve the detectability of low-contrast lesions. So SNR calculated in this way cannot be used to compare lesion detectability in images reconstructed using different filters or voxel sizes. SNR is, of course, considerably easier to measure than NEC.

We have identified expressions for predicting the activity required to achieve the target value for NEC_patient_ in terms of sex, height and weight and have proposed a method for determining the activity to be administered and the acquisition time per bed position in terms of these easily measurable parameters. This should yield images of consistently appropriate quality with reduced patient and staff doses and practical scanning times.

Figure 3 illustrates why increasing the administered activity is less effective at improving image quality than the same proportional increase in acquisition time, particularly for heavier patients for whom the NECR may already be approaching its peak value. In this case, the length of patient scanned by bed positions between the top of the bladder and the shoulders was 0.47 m, so the NEC required to achieve the target value for NEC_patient_ is 17 Mcounts (37 × 0.47). An administered activity of 237 MBq gives an NECR of 4.35 Mcount/min. In 3 min, this will give an NEC of 13 Mcounts, 75% of the required value. The target NEC can be achieved by increasing the acquisition time by 33% to 4 min per bed position. However, to achieve the same increase in NEC by increasing the administered activity, the NECR must be increased by 33% to 5.80 Mcounts/min. The curvature of the plot of NECR against administered activity means that this requires an activity of 365 MBq, an increase of 54%.

de Groot et al. [4] have found that if the activity administered is scaled according to the square of the subject’s weight, SNR_liver_ is independent of the subject weight, and this is the basis for the quadratic expressions for the administered activity and administration time included in the current EANM Guideline [8]. The expression they derived for maintaining constant image quality did not include a height term.

Accorsi et al. [12] used a target NEC_density_ based on the value obtained using a standard protocol for a 70-kg adult to derive expressions for scaling the administered activity and acquisition times for paediatric patients according to the weight of the patient.

By contrast, we have found that to achieve our target for NEC_patient_, activity should be scaled according to height^− 0.93^ × weight^1.69^ for smaller subjects scanned at 3 min per bed position and height^− 0.77^ × weight^1.54^ for larger subjects scanned for 4 min per bed position. The inclusion of the height term means that for two subjects of the same weight, the taller subject will require less activity to achieve the same NEC_patient_.

**Fig. 6:**
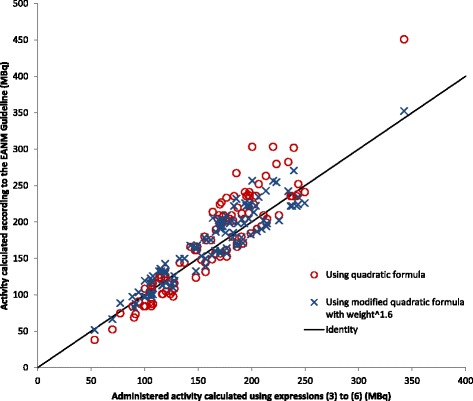
Administered activity calculated using the quadratic formula described in the current EANM Guideline plotted against activity calculated using expressions (3) to (6)

When the analysis described by de Groot et al. was repeated on this dataset, we found that the administered activity should be scaled to weight to the power of 1.6, rather than 2 in order to achieve SNR_liver_ independent of the subject weight. Figure 6 shows the activity to be administered to each subject according to the quadratic formula described in the current EANM Guideline for a bed overlap of >30% using acquisition times calculated using expressions (3) to (6), plotted against activity calculated using expressions (3) to (6). Also shown are activities calculated using the formula from the EANM guideline modified by changing the power to which the patient weight is raised from 2 to 1.6. The close agreement between the later set of values and those calculated using expressions (3) to (6) arises because the power to which weight is raised in expressions (4) and (6) is close to 1.6 but also reflects the fact that the administered activity and acquisition time required for this scanner to achieve the target image quality identified in this study for a typical 75-kg patient coincides with those recommended in the EANM Guideline. This would not be the case using a scanner with a different sensitivity. For example, had we used a scanner without an extended axial field of view and with a lower sensitivity, the activities and/or acquisition times needed to achieve the target NEC_patient_ would have been higher. The formulae for administered activity and administration time in the current EANM Guideline do not take into account differences in scanner sensitivity, although this is under review by EANM Research Ltd (EARL) [19].

The mean activity that would have been administered to the subjects of this study using expressions (3) to (6) is 66 MBq (29%) lower than was actually administered using the current protocol and 11 MBq lower than the mean activity recommended using the quadratic formula described in the current EANM Guideline calculated as described above.

### Further work

Validation is required to check that using the expressions proposed for administered activity and scan duration does result in PET images of consistent quality with a reduction in average administered activity and scan duration compared to the current protocol.

This work identified a protocol for a particular scanner with an extended axial field of view and TOF and a particular reconstruction protocol for use for patients referred for whole-body ^18^F-FDG scans for a range of indications. The work needs to be repeated at other centres on different types of scanner and using different reconstruction protocols. Protocols could be developed for specific indications and regions of the body. However, it might be possible to modify the protocol developed here for different scanners and applications using a measure of NECR or other phantom-based measures of system sensitivity similar to that being evaluated by EARL [19].

The expressions proposed will result in administered activities which are consistent with the DRL specified by ARSAC applicable to centres in the UK [6]. Before this protocol is implemented in centres in other countries, they would need to check that it is consistent with their national regulations.

## Summary

NEC_patient_ was identified as the objective measure which best agreed with the physician’s assessment.

37 Mcounts/m was identified as a target NEC_patient_.

Expressions in terms of patient height and weight have been identified to determine the administered activity and scan time per bed position required to achieve images of consistent quality for patients with varying body size and composition.

These expressions require validation and might need to be adapted for different scanners and reconstruction parameters.

## Additional file

Additional file 1: Image quality data and data used to derive equations (3) to (6). (XLSX 83.5 kb)
